# Active Disaster Response System for a Smart Building

**DOI:** 10.3390/s140917451

**Published:** 2014-09-18

**Authors:** Chun-Yen Lin, Edward T.-H Chu, Lun-Wei Ku, Jane W. S. Liu

**Affiliations:** 1 Department of Computer Science and Information Engineering, National Yunlin University of Science and Technology, Douliou, Yunlin 64002, Taiwan; E-Mail: u9917010@yuntech.edu.tw; 2 Institute of Information Science, Academia Sinica, Taipei 11529, Taiwan; E-Mails: lwku@iis.sinica.edu.tw (L.-W.K.); janeliu@iis.sinica.edu.tw (J.W.S.L.)

**Keywords:** intelligent system, disaster preparedness, smart environment, automatic response system

## Abstract

Disaster warning and surveillance systems have been widely applied to help the public be aware of an emergency. However, existing warning systems are unable to cooperate with household appliances or embedded controllers; that is, they cannot provide enough time for preparedness and evacuation, especially for disasters like earthquakes. In addition, the existing warning and surveillance systems are not responsible for collecting sufficient information inside a building for relief workers to conduct a proper rescue action after a disaster happens. In this paper, we describe the design and implementation of a proof of concept prototype, named the active disaster response system (ADRS), which automatically performs emergency tasks when an earthquake happens. ADRS can interpret Common Alerting Protocol (CAP) messages, published by an official agency, and actuate embedded controllers to perform emergency tasks to respond to the alerts. Examples of emergency tasks include opening doors and windows and cutting off power lines and gas valves. In addition, ADRS can maintain a temporary network by utilizing the embedded controllers; hence, victims trapped inside a building are still able to post emergency messages if the original network is disconnected. We conducted a field trial to evaluate the effectiveness of ADRS after an earthquake happened. Our results show that compared to manually operating emergency tasks, ADRS can reduce the operation time by up to 15 s, which is long enough for people to get under sturdy furniture, or to evacuate from the third floor to the first floor, or to run more than 100 m.

## 1. Introduction

Disaster warning and surveillance systems have been widely used by official agencies to send to the public emergency information before disasters strike. In addition to recent advances in technologies for the prediction and detection of disasters and ICT (Information and Communications Technology) support infrastructures for the distribution of warnings messages, international emergency data exchange language standards have been adopted for encoding alert messages. For example, the XML-based standard, Common Alert Protocol (CAP) [1], has been adopted in the USA, Canada, Australia and parts of the Asian Pacific region, including Taiwan and Japan. Official agencies in these regions can now generate accurate warnings of many types of natural disasters a few seconds or minutes before they occur. The warning messages are encoded in CAP format and broadcasted to the public, so that people can stay away from harm. For example, in Taiwan, CAP-formatted warning messages issued by the Central Weather Bureau, the Water Resource Agency, Soil and Water Conservation Bureau and the Directorate General of Highways are converted by Google Public Alert [2] into short messages and delivered to mobile phone users via the Google Now personal assistant. Although the public can get warning messages issued by official agencies, the warnings are not utilized as effectively as they can be today. CAP alert messages, though machine-readable, are consumed mostly by people to date. Limitations in human reaction time can significantly limit the effectiveness of the warnings. A better alternative is to deliver the messages directly to smart devices and applications that can respond with humanly impossible speed and to let them take appropriate actions to keep people away from any potential danger and their belongings from being damaged.

Because earthquakes hit Taiwan frequently, we designed and implemented an active disaster response system (ADRS) to respond to earthquakes. Due to great advances in geotechnical earthquake engineering, a modern earthquake early warning system (EEWS) can determine the magnitude and the location of an earthquake in three seconds after an initial P-wave has been detected [3,4]. This computation time is expected to get shorter and shorter in the near future. In Taiwan, strong motion monitors deployed densely can detect a significant earthquake and determine the affected area almost instantaneously after an earthquake occurs. According to the report of the National Center for Research on Earthquake Engineering (NCREE), in Taiwan, it takes 30 s for earthquake shock waves to propagate from Taichung (the middle of Taiwan) to Taipei (the north of Taiwan). The distance between the two places is around 150 km. An EEWS has already been deployed and proved that it can provide a warning to Taipei 27 s prior to the arrival of shock waves after an earthquake, such as 921 earthquakes [5], happening in the middle of Taiwan. With ADRS, there will be enough time for people to move to safe places, to put sensitive equipment into a safe mode and to automatically open elevator doors before the major shaking occurs.

In order to realize the abovementioned idea, we designed and implemented a proof of concept prototype, named the active disaster response system. We implemented ADRS by Tibbo EM1000TEV microcontrollers [6], which can interpret standardized alert messages published by official agencies and automatically perform emergency tasks when an earthquake is expected to strike in order to prevent the loss of lives, to reduce the chance of injuries and to minimize economic losses. For example, ADRS will shut off natural gas intake valves to prevent fire, open escape doors to ease evacuation, bring elevators to the nearest floor to evacuate trapped people, turn off electric appliances to avoid electrical fire, and so on, when receiving a strong earthquake alert before ground movement begins. In addition, after an earthquake, ADRS utilizes the embedded controllers to maintain a temporary network so that victims trapped inside a building can post emergency messages, even when the normal network connections are disrupted. We conducted a field trial to evaluate the effectiveness of ADRS when it received a strong earthquake alert. Our results show that compared to manually operating emergency tasks, ADRS can reduce the operation time by up to 15 s, which is long enough for people to get under sturdy furniture, or to evacuate from the third floor to the first floor, or to run more than 100 m.

The rest of this paper is organized as follows. Section 2 reviews related work to justify and motivate our work. Section 3 describes the design and implementation of ADRS. Section 4 presents the evaluation results. Finally, Section 5 concludes this work with future directions.

## 2. Related Work

In this section, we first explore related works in the field of disaster warning systems and disaster surveillance systems. We then discuss several works related to disaster information collection.

### 2.1. Disaster Warning System

Disaster warning systems are used to broadcast warning messages to the public and, thus, warn people of imminent disasters. Sing *et al.* [7] developed a seismic early warning alert system that can forewarn an urban area of a forthcoming strong quake, so that people could take appropriate actions and respond to that situation effectively. Rahman *et al.* [8] developed a warning system, named OpenStreetMap (OSM), which provides a specific service to notify people about warning information and to help them determine the shortest path for evacuation. Wijesinghe *et al.* [9] utilized GSM information to build a disaster early warning network and adopted the network to deliver warning messages. When the proposed disaster warning system receives alert messages from the warning network, it will make a loud noise, turn on warning lights and broadcast messages to notify people. Azmi *et al.* [10] proposed a disaster emergency warning system based on the DVB-T (Digital Video Broadcasting-Terrestrial) standard. Adam *et al.* [11] and Chu *et al.* [12,13] used social media, such as Twitter and Facebook, to collect disaster information. Based on the collected information, they then broadcasted warning messages to registered users through smart phones. Although these warning systems can send alerts to the public, none of them adopted CAP standards for encoding alert messages. Because their warning messages are not machine-readable, these warning systems are incompatible with smart devices to take appropriate actions to keep people away from potential danger. ADRS is a further extension of our previous research on cyber-physical elements of a disaster-prepared smart environment [14,15]. Compared to our previous work, this paper mainly focuses on the design and implementation of a proof of concept prototype to automatically perform emergency tasks when an earthquake happens.

### 2.2. Disaster Surveillance System

In order to collect disaster information promptly, some research efforts have been devoted to developing disaster surveillance systems. Di Martino *et al.* [16] utilized radar monitoring of geographic information to assess the damage of a natural disaster to support a rescue decision. Lu *et al.* [17] adopted virtual human resources (VHR) to get ground information and to evaluate the damage of collapsed buildings. However, their system cannot collect the information about trapped victims inside a building. Similarly, Post *et al.* [18] used Earth observation and modeling technologies to monitor disaster damages. Although their system estimates the threatened areas of some natural disasters, such as landslide or flooding, it cannot provide detailed information on the affected people. In short, all of the above methods cannot provide detailed information about victims trapped inside a building. In order to address this problem, ADRS utilizes embedded controllers to maintain a temporary network, as stated earlier. Based on our design, victims can post an emergency message to an embedded controller through a one-hop network. Therefore, they have a good chance of being found in the one-hop transmission range.

### 2.3. Disaster Information Collection

Some crowdsourcing strategies have been developed to collect disaster information. Vivacqua *et al.* [19] proposed a collective intelligence technique to manage disaster information. Yin *et al.* [20,21] first utilized social media to quickly gather information about disaster situations from people close to threatened areas. They then used the collected information to help relief workers to conduct rescue actions. Shan *et al.* [22] and Seop *et al.* [23] adopted smart phones to collect disaster information. People can upload pictures, videos and text messages to a remote server to report damage to the environment. Farber *et al.* [24] adopted disaster portals to collect disaster messages via the Web 2.0 social network. The collected information is used to help command centers to gather disaster information rapidly. Unlike existing works, ADRS utilizes embedded controllers inside a building to maintain a temporary network for trapped victims.

## 3. Active Disaster Response System (ADRS)

In this section, we first give an overview of ADRS. We then describe the emergency broadcast system and active disaster response system. Finally, several usage scenarios of ADRS are discussed.

### 3.1. Overview of ADRS

**Figure 1.**
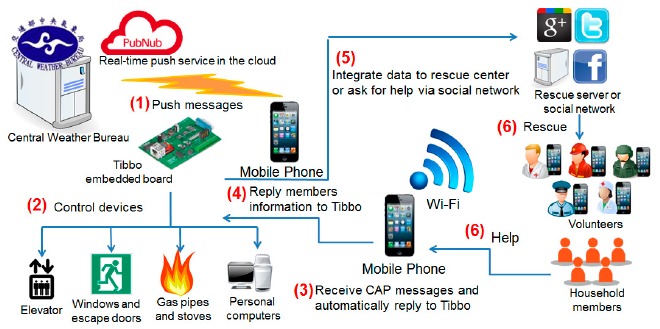
Active disaster response system flow chart.

Figure 1 shows a use scenario of ADRS. When a natural disaster happens, the Central Weather Bureau (CWB) publishes CAP warning messages. The messages are received and interpreted by Tibbo embedded boards inside a building (Step 1). If a warning message indicates that a strong earthquake is coming, these components of ADRS will automatically perform emergency tasks, such as opening doors, cutting off power and gas, and so on (Step 2), in order to prevent the loss of lives, to reduce the chance of injuries and to minimize economic losses. After the earthquake, ADRS configures Tiboo EM1000-TEV embedded boards [6] to construct a temporary network (Steps 3 and 4). After the network connections to the outside world are recovered, Tibbo will deliver these messages to relief workers nearby or the server of a command center, so that rescue actions can be well planned (Steps 5 and 6).

### 3.2. Emergency Broadcast System

**Figure 2.**
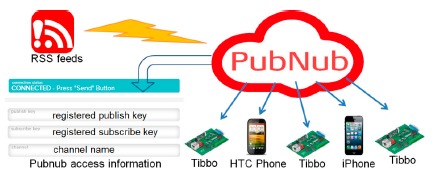
Deliver messages via PubNub.

In our prototype, we designed and implemented an emergency broadcast system that simulates the Central Weather Bureau to publish warning messages. We adopt PubNub's real-time push service [25] to push warning messages to Tibbo devices and smart phones in a building. As Figure 2 shows, PubNub can deliver hundreds of thousands, even millions, of real-time messages to devices at the same time by utilizing cloud infrastructure and services. In our implementation, we used a smart phone to act as an emergency broadcast system that publishes disaster warning messages. For this, we first registered a PubNub account to get its own subscribe key and publish key. We then used the publish key and the subscribe key to publish and retrieve warning messages with PubNub APIs (Application Programming Interfaces), shown in Table 1.

**Figure 3.**
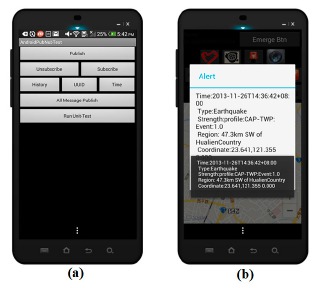
Deliver messages via PubNub. (**a**) PubNub broadcast; (**b**) PubNub receiver.

Figure 3a shows the user interface of the emergency broadcast system on an Android smart phone. This mobile emergency app (application) provides key functions to publish warning message, such as publish, subscribe, unsubscribe, history records, and so on. Figure 3b shows a warning message about an earthquake received by an end user.

### 3.3. Active Disaster Response System

The active disaster response system consists of three key components: CAP message parser and actuators, emergency message board and emergency report system.

#### 3.3.1. CAP Message Parser and Actuators

**Figure 4.**
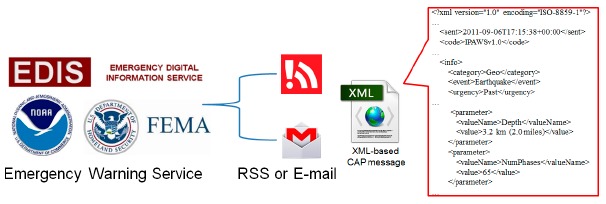
CAP messages published by an early warning system (EWS). EDIS, Emergency Digital Information Service.

In addition to our simulated emergency broadcast system, ADRS registers for the CAP messages provided by the Emergency Digital Information Service (EDIS), the National Science and Technology Center for Disaster Reduction (NCDR) [26] and the Integrated Public Alert and Warning System (IPAWS). As shown in Figure 4, there are two ways to get warning messages. One is from RSS feeds, and the other is from email. IPAWS of NCDR sends XML-based CAP messages by email or RSS to registered receivers.

**Figure 5.**
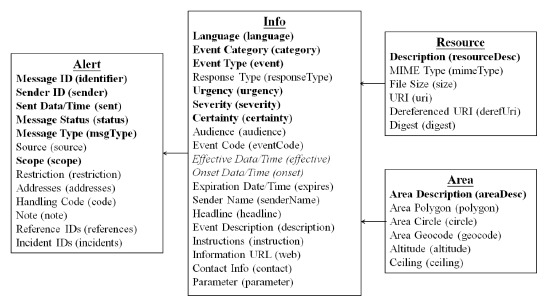
Common alerting protocol architecture.

Figure 5 shows the major four parts in a Common Alerting Protocol message. They are alert, info, resource and area. The CAP parser extracts important information from a message, respectively. First, the CAP parser extracts message ID (Identification), sender ID, sent data, and so on, from the alert part. Second, the CAP parser extracts event category, event type, urgency, and severity and so on from the info part. Third, the description is extracted from the resource part. Finally, the CAP parser extracts area description from the part of area. The Common Alerting Protocol Validator (CAPV) [27] is used to validate the correctness of our CAP parser.

**Figure 6.**
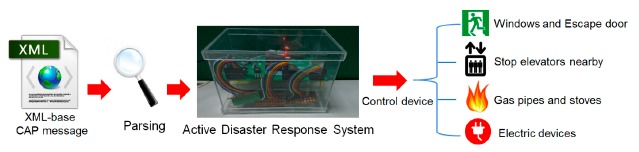
The flow of parsing CAP messages and controlling devices.

Figure 6 shows the flow of parsing CAP message and controlling associated devices. After parsing a CAP alert message warning of an imminent earthquake, ADRS obtains earthquake information, including urgency, severity, magnitude, depth, *etc.* According to the standard operating procedure (SOP) for earthquakes, in order to reduce potential dangers, people who are inside a building should open doors and windows, cut off gas and turn off light and power before evacuating to a safe place [28,29]. Therefore, we designed ADRS to automatically perform these tasks, so as to help people to evacuate faster and to keep their belongings from being damaged.

ADRS needs to be able to verify that a CAP message it receives was issued by an authorized source. As CAP is an XML-based format, existing XML security mechanisms can be used to secure and authenticate the content of a CAP message. The commonly used security mechanism is described as follows. First, the receiver has to ensure that the CAP message was delivered from a trustworthy entity, such as a trusted SIP (Session Initiation Protocol) proxy, and that the communication channel between the receiver and the SIP proxy is properly secured. Second, the sender of the CAP message should be on the whitelist. Finally, the message should be protected by a digital signature, and the entity signing the CAP message should also be listed on the whitelist. If none of these verification checks indicates a known sender, the CAP message should be treated as malicious and suspicious. As mentioned in Section 3.2, we used PubNub real-time push service to push CAP messages to receivers. We first registered a PubNub account to get a subscribe key and a publish key. We then used the two keys to establish a secure channel between the sender and the receiver.

#### 3.3.2. Emergency Message Board

According to the historical records and statistics [30,31], massive earthquakes usually cause power outages lasting three hours or longer. During this time, connections to the outside of the building, including cell phone connections, may be disrupted. Therefore, ADRS utilizes the embedded controllers to maintain a temporary network, as stated earlier. In this work, Tibbo EM1000-TEV embedded boards were used to build up a prototype of a one-hop network.

**Figure 7.**
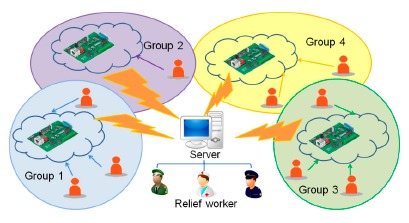
Usage scenario of emergency message board.

**Figure 8.**
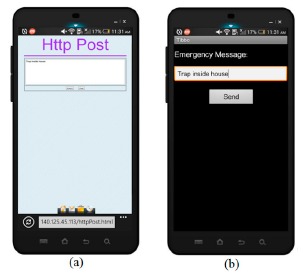
Emergency message interface: (**a**) web interface; (**b**) Android interface.

Table 2 shows the hardware specification of the Tibbo EM1000-TEV device. The core of the board is a RISC (Reduced Instruction Set Computing) chip with 88 MHz. It supports both Wi-Fi and Ethernet interfaces. It also has a 2-KB EEPROM (Electrically Erasable Programmable Read Only Memory) to store important information. Figure 7 shows a use scenario of the emergency board. There are several embedded boards inside a building. Each of them has network capacity. When the Internet is disrupted, people can be connected to these embedded boards directly and post messages on emergency events. The events are stored in the embedded boards. As Figure 7 shows, people or victims may be divided into several groups, depending on their locations. When relief workers come to the threatened area, they can get emergency reports from these embedded boards and plan rescue actions. Figure 8 shows the two kinds of user interfaces of the emergency message service: the web interface and the Android app interface. Victims can use one of the interfaces to post emergency messages on the embedded boards. Since embedded boards are usually powered by battery, it is desirable to reduce possible communication to save energy. As a result, for victims, it is necessary to properly select an embedded board to store their emergency messages.

In order to address this issue, a heuristic algorithm, called Algorithm 1, was designed to select an embedded board to store emergency messages. For each victim *i*, *m_i_* denotes the size of emergency message needed to be posted to a Tibbo embedded board. Furthermore, *e_i_* is the approximated energy consumption of receiving the emergency message. In the beginning, the victim searches embedded boards nearby and queries the remaining energy capacity and memory capacity of each embedded board. Let *k* denote the number of Tibbo boards nearby victim *i*. The remaining energy capacity of the *k*-th Tibbo is *E_k_* and its remaining memory capacity is *M_k_*.

Algorithm 1 shows the method to post emergency messages. The post function is used to select a suitable Tiboo board to post the emergency message. The goal is to reduce energy consumption as much as possible when posting emergency messages. For this, Algorithm 1 examines all Tiboo boards near the victim *i* (Lines 3 to 9). If the remaining energy capacity of the Tiboo board is larger than *e_i_* and its remaining memory capacity is larger than *m_i_*, it will be selected as a candidate. The post function picks up one embedded board among the candidates with the highest energy as the result (Lines 5 and 7). After selecting the target embedded board, the emergency message is posted to the board. The advantage of Algorithm 1 is the ease of implementation, and it can be integrated with exit signs and emergency lights. When official responders arrive at the threatened area, they can connect to these embedded boards to obtain emergency requests.



**Algorithm 1: Post emergency messages**
**Input:**
*m_i_*, *e_i_*, *k*, *E_k_*,*M_k_*,*m_i_*:the size of emergency the message needed to be posted*e_i_*:the approximated energy consumption of receiving the emergency message.*k*:the number of Tibbo nearby victim *i* is *k*.*E_k_*:the remaining energy capacity of the *k*-th Tibbo*M_k_*:the remaining memory capacity of the *k*-th TibboS:the selected Tibbo board
1:Procedure **Post**2:S = Null;3:**for**
*j* = 1 to *k*
**do**4: **if** (S == Null) AND (*E_j_* > *e_i_* ) AND (*M_j_* > *m_i_*
**) then**5:  S = *j*;6: **else if** (S! = Null) AND (*E_j_* > *E_s_* ) AND (*M_j_* > *m_i_* ) **then**7:  S = *j*;8: **end if**9:**end for**10: **if** S is not Null11:  Connect to Tibbo board S;12:  Post the emergency message on S;13:**end if**


#### 3.3.3. Emergency Report System

**Figure 9.**
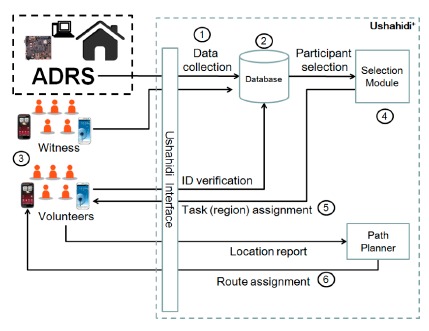
The relationship between ADRS and a crowdsourcing-enhanced emergency management system.

The emergency report system is designed to send collected disaster information to the responsible emergency response agency for further use. Figure 9 shows how ADRS reports emergency events to the platform Ushahidi^+^ [26], which is an extension of Ushahidi. As Figure 9 shows, when a disaster happens, the disaster information is first collected by official responders, ADRS, witnesses or victims (Step 1). The responsibility of ADRS is to collect victims' information through sensors deployed in buildings and to forward the information to Ushahidi^+^. The collected disaster information is stored in the database of Ushahidi^+^ for the threat level or threatened areas analysis (Step 2). The original Ushahidi classified events into eight types: emergency, vital lines, public health, security threats, infrastructure damage, natural hazard, services available and others. In the implementation, system administration should assign a score to each type of event to indicate its emergency level. If the threat level or the threatened area is very serious, the official agency will post volunteer recruitment messages on a social network, such as Facebook or Twitter, in order to attract more volunteers to improve rescue actions (Step 3). People who want to volunteer for rescue actions can report their personal information, such as name, location and professional specialty, to the official agency by an emergency app or Ushahidi^+^ web interface. After recruiting enough volunteers, the official agency then triggers the participant selection process in order to supplement surveillance sensor coverage (Step 4). The allocation results are sent to volunteers (Step 5). Different volunteers may be assigned to different regions to collect different disaster information. After volunteers arrive at the target region, the path planner then calculates a precise route to the destination for each volunteer (Step 6). Finally, all information collected by the volunteers is sent back to the official agency for further analysis. For readers who are interested in Ushahidi^+^, please refer to our previous work [12,13].

### 3.4. Prototype of ADRS and Use Scenarios

**Figure 10.**
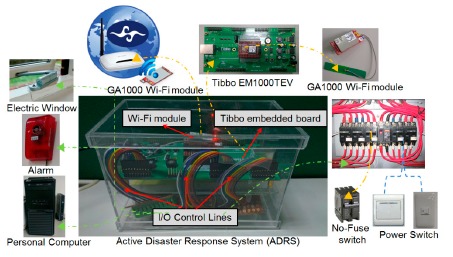
Prototype of ADRS.

Figure 10 shows the prototype of ADRS, which consists of three parts of the Tibbo development kit. They are the input/output control lines and the Wi-Fi SPI module. Tibbo boards are used to receive warning messages published by the Central Weather Bureau and parse the XML-based format CAP messages to extract disaster information. In addition, a Wi-Fi SPI module is adopted so as to provide a wireless network function to Tibbo, which can also collect household members' messages. These messages are stored in the flash memory. Because a user can connect to a Tibbo in one hop to post emergency messages, he or she, if trapped, will be found in the transmission range of one hop, which is around 20 to 30 m. The last part of ADRS is the input/output control lines, which are used to control relay switches, so that home devices can be turned on or off and opened or closed. In our prototype, Tibbo can control alerts, doors, power, gas, *etc.*, or even use Wi-Fi wireless networks to control devices, like personal computers, to save important data. We installed ADRS in the smart home demo room (EB208) in the CSIE (Computer Science and Information Engineering) department at National Yunlin University of Science and Technology, Taiwan.

**Figure 11.**
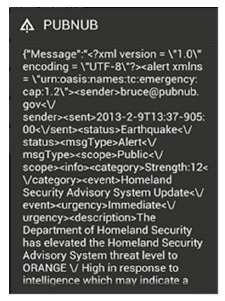
CAP message.

**Figure 12.**
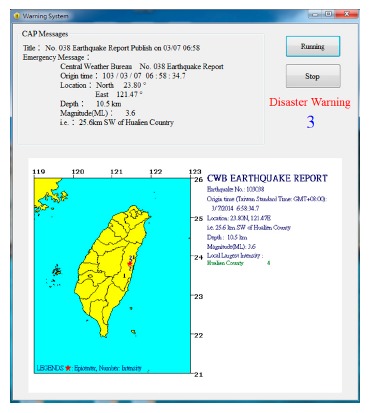
Human readable version.

Figure 11 shows a CAP message containing an earthquake alert delivered by the PubNub service. After receiving the warning message, ADRS converts it to a human readable version for people in the building (shown in Figure 12) and performs associated emergency tasks.

Please note that ADRS should go through a rigorous testing before it can be deployed. In order to address the issue of power failure, in our prototype, the ADRS connects to a UPS (uninterruptible power supply) that provides emergency power when the input power source fails. In addition, the electric door lock we adopted can be unlocked by remote (*i.e.*, ADRS) or by hand. When the unlock button of the electric door is pressed, the power of the control circuit is cut, so that the remote cannot prevent the door from being opened.

We note that ADRS, in addition to earthquakes, can process alerts for other types of disasters and be extended to respond to them in and out of buildings. For example, when landslides and debris flows happen, ADRS can trigger message signs put up before tunnels and bridges to warn drivers to slow down and pull over. For flash floods, ADRS can change the traffic light to red, so that the roads can be closed. It also can turn on pumps to extract floodwater or start automatic flood barriers, to save low-lying buildings from rising water levels. For tornados, ADRS can automatically open the outside doors and vents to equalize air pressure inside and outside of a house, preventing it from exploding and also unlock each shelter door to minimize the chance of such a tragedy. For volcanic eruptions, ADRS can cooperate with microcontrollers to close all windows and doors to protect people from volcanic ash.

ADRS should be able to deal with false negatives, which are false alarms due to system errors. Because CAP messages are also posted on authenticated websites, such as NCDR in Taiwan [26], we can adopt a two-way communication to avoid false alarms induced by system software errors. ADRS, before performing emergency tasks, should use the hyperlink in the alert segment of the received CAP message to connect to the authenticated website and search for the same CAP message. If the same CAP message cannot be found, ADRS should cancel the emergency tasks to avoid false alarms. In addition, performing routine maintenance on the hardware devices is also needed to avoid possible false alarms caused by system hardware errors. ADRS also should be able to deal with false negatives when the system fails to respond. Among the solutions, one of the practical ways is to use an external watchdog timer to detect the status of ADRS. During normal operation, ADRS regularly resets the watchdog timer to prevent it from timing out. If, due to a hardware fault or program errors, ADRS fails to restart the watchdog, the timer will elapse and generate a timeout signal. The timeout signal is then used to reset ADRS or to perform predefined tasks, such as sending a warning message to the person who is in charge of the maintenance of ADRS. We consider the above mechanism used to deal with false positives and false negative as future work.

## 4. Evaluation Results

This section first presents the performance of ADRS obtained in a series of experiments to evaluate ADRS's effectiveness at reducing operation time and evacuation time. It then presents a performance evaluation of the emergency message board.

### 4.1. Reduction in Operation Time

**Figure 13.**
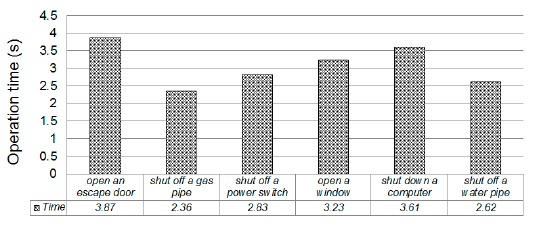
The operation time of each task.

According to the standard operating procedure (SOP) for earthquake responses, people who are inside a building should open doors and windows, cut off gas and turn off lights and power before evacuation [28,29]. Opening windows and doors can prevent the doors or the windows from being jammed by the large deformation caused by an earthquake and can ensure that people are not trapped inside. Another reason for leaving doors and windows open during and after an earthquake is to avoid possible fires or explosions due to a gas leakage inside the building. In addition, cutting off natural gas valves can avoid a possible fire or explosion due to a gas leakage. Furthermore, when a massive earthquake happens, fire can induce thick smoke and cause serious damage. Finally, electrical appliances or computers should be shut off or put into a safe mode in order to avoid possible flames, fire and loss of data. We designed ADRS to automatically perform these important tasks so as to help people evacuate faster and to keep their belongings from being damaged. In order to investigate the effectiveness of ADRS, we first measured the time needed to perform these tasks manually. The operation time of each task includes two parts. The first part is completion time, which is the time needed to complete the task. The second part is the response time, which is the time interval moving from the location of a control device to the location of another device. The response time represents human reaction time. In our experiment, we set the response time to one second. To measure the completion time of a task, we asked eight volunteers to manually perform the task and then found the average. Figure 13 lists the operation time of each task. For example, opening a door takes 3.87 s, opening a window 3.23 s and shutting down a computer 3.61 s. It requires a total of 18.52 s to do all of earthquake tasks manually. With the support of ADRS, all of these tasks are automatically and simultaneously finished in three seconds. As a result, ADRS can reduce the operation time by up to 15 s, which is long enough for people to get under sturdy furniture, or to evacuate from the third floor to the first floor, or to run more than 100 m.

### 4.2. Effect on Evacuation Time

The standard operating procedure for earthquakes also tells people to first evacuate to a safe place if they are on a lower floor [28,29]. They should stay away from buildings and trees to avoid potential injury. On the other hand, people on a higher floor should first find protection in a doorway or crouch under a desk or table. They then should evacuate to a safe place after the shaking becomes small. ADRS can play an important role in both scenarios to help people evacuate.

**Figure 14.**
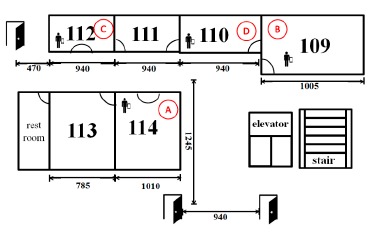
Experiment environment.

**Figure 15.**
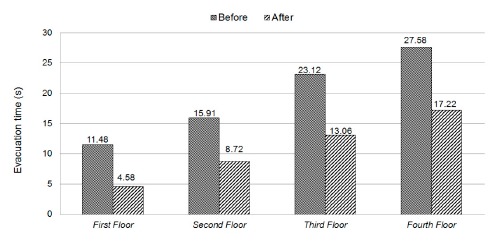
Comparison of evacuation time.

In this experiment, we investigated the effect of ADRS on evacuation time. We first asked several volunteers to evacuate from different floors inside a building to a safe place outside the building. The experiment was conducted at the Engineering Building V of National Yunlin University of Science and Technology, which is a four-floor building. The floor plan of each floor is similar to that of the first floor, which is shown in Figure 14. In our experiment, we repeated three rounds for each floor. For each round, we asked a volunteer to follow a certain path to evacuate from a floor to a safe place outside the building. Volunteers on the third or the fourth floor were asked to shut down a computer, open windows and an escape door. On the other hand, volunteers on the first and second floors needed to open windows and an escape door only. We measured the time taken by volunteers to evacuate from different floors and present the average time in Figure 15. We found that ADRS can reduce evacuation time from 6 to 10 s, which is long enough for faculty or students to get under sturdy furniture, or to evacuate from the second floor to the first floor, or to run more than 50 m.

### 4.3. Performance Evaluation of Emergency Message Board

**Figure 16.**
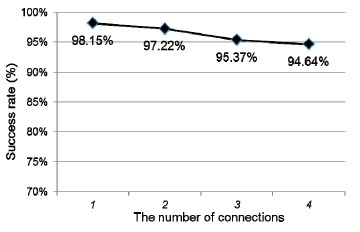
Stability of emergency message board.

ADRS utilizes Tiboo embedded boards to maintain a temporary network, so that victims trapped inside a building can still post emergency messages to a Tiboo embedded board when connections to the outside are disrupted. This experiment studies the effect of the number of connections on the success rate of packet transmission. The size of a message is 20 bytes, which represents a short emergency message in a real scenario. For each experiment, we repeat the transmission 100 times. The average results are shown in Figure 16. As is shown, the Tiboo embedded board can maintain a highly success rate of packet transmission. When the number of connection increases to four, the success rate slightly decreases to 94.64%. These figures indicate that ADRS can be applied to real scenarios for practical use.

**Figure 17.**
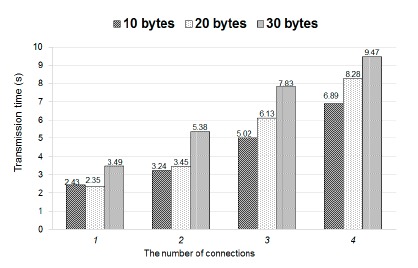
Estimate transmission time.

In addition, we conducted an experiment to evaluate the effect of node failure on transmission time. Figure 14 shows the experimental environment, in which “A”, “B”, “C” and “D” represent the embedded boards, respectively. Each smart phone can connect to each embedded board in one hop. When a node fails, the original connection to the failure node should be rebuilt on other nodes, thus increasing the traffic on the affected nodes. Therefore, we investigated the effect of the number of connections on transmission time to simulate the scenario of node failure. The results are shown in Figure 17, in which the *x*-axis represents the number of connections on Node A. The more failure nodes there are, the higher the number of connections on Node A there is. Due to the sufficient network bandwidth, we found that the transmission time increases slightly when the number of connections increases. We also measure the transmission time of three different sizes of emergency message. These are 10-byte, 20-byte and 30-byte emergency messages. Our results show that the transmission time remains almost the same as long as the size of an emergency message is more than 20 bytes and the number of connections is small. For example, when the number of connections becomes two, the transmission time of a 10-byte emergency message is 3.24 s and that of a 20-byte emergency message 3.45 s. Our results also show that emergency messages can be posted to a node in 10 s in the experiments we have tested.

## 5. Conclusions

The ADRS described in this paper was designed to automatically perform emergency tasks when a strong earthquake is expected to strike in seconds in order to prevent the loss of lives, to reduce the chance of injuries and to minimize economic losses. We realized the idea by using Tibbo EM1000TEV embedded boards to receive CAP earthquake warning messages published by the Central Weather Bureau, Taiwan, and to parse the messages to extract all of the information about the earthquake. Based on the extracted information, ADRS then controls microcontrollers to perform the corresponding emergency tasks. After the earthquake strikes, ADRS constructs a temporary network to collect emergency messages posted by trapped victims, to save the messages and to deliver the messages to command centers when connections to the outside world are reestablished. Compared to the manual operation of emergency tasks, ADRS can reduce the operation time by up to 15 s, which is long enough for people to get under sturdy furniture, or to evacuate from the third floor to the first floor, or to run more than 100 m, which indicates that ADRS can be applied to real scenarios for practical use. In the future, we plan to further enhance the functionality of ADRS to handle false alarms and the conditions when the system fails to respond.

## Figures and Tables

**Table 1.** PubNub APIs [25] used in the active disaster response system (ADRS).

**API name**	**Description**
PubNub	To create PubNub service, put parameters as publish_key and subscribe_key
Publish	To publish messages, put parameters as channel_name, messages
Subscribe	To subscribe messages, put parameters as channel_name, callback function

**Table 2.** Tibbo EM1000-TEV hardware specification.

Tibbo EM1000-TEV Specification
1. 88 MHz RISC (Reduced Instruction Set Computing) chip (T1000)
2. 10/100 BaseT auto-MDIX Ethernet
3. Provide Wi-Fi interface (GA1000 module)
4. Four high speed serial port (CMOS)
5. 1024-KB flash memory for firmware and application
6. 2-KB EEPROM (Electrically Erasable Programmable Read Only Memory) for parameters and data storage
